# Laparoendoscopic rendezvous procedure in a patient with situs inversus totalis: A case report and review of the literature

**DOI:** 10.1002/ccr3.3240

**Published:** 2020-08-11

**Authors:** Alejandro Brañes, Gustavo Pérez

**Affiliations:** ^1^ Digestive Surgery Department School of Medicine Pontificia Universidad Católica de Chile Santiago Chile

**Keywords:** choledocholithiasis, cholelithiasis, endoscopic retrograde cholangiopancreatography, laparoscopic cholecystectomy, Rendezvous, situs inversus

## Abstract

Situs inversus totalis patients may be associated with difficulties in the diagnosis and treatment of surgical diseases. This case suggests that laparoendoscopic rendezvous procedure could be associated with a lower morbidity and length of hospital stay.

## Graphical abstract

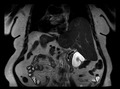

## 1 INTRODUCTION

Situs inversus totalis (SIT) is a rare congenital condition present in 1:10 000 to 1:20 000 cases,^1^ in which thoracic and abdominal organs are arranged in a mirror image of their normal position.^2^ This disorder generates additional challenges to the treatment of surgical pathologies as normal anatomy is entirely modified. The frequency of cholelithiasis in these patients seems to be similar to the general population.^3^ Therefore, the frequency of bile duct disease, such as choledocholithiasis, may be similar as well. In previous reports, these patients have been treated mainly by a laparoscopic cholecystectomy (LC) and preoperative or postoperative endoscopic retrograde cholangiopancreatography (ERCP) as a two‐stage procedure or laparoscopic cholecystectomy and laparoscopic common bile duct exploration (LC + LCBDE) as a one‐stage procedure. To our knowledge, this is the first case reported in literature of a patient with SIT with associated cholelithiasis and choledocholithiasis treated by a laparoendoscopic rendezvous (LER) technique.

## 2 CASE PRESENTATION

**Figure 1:**
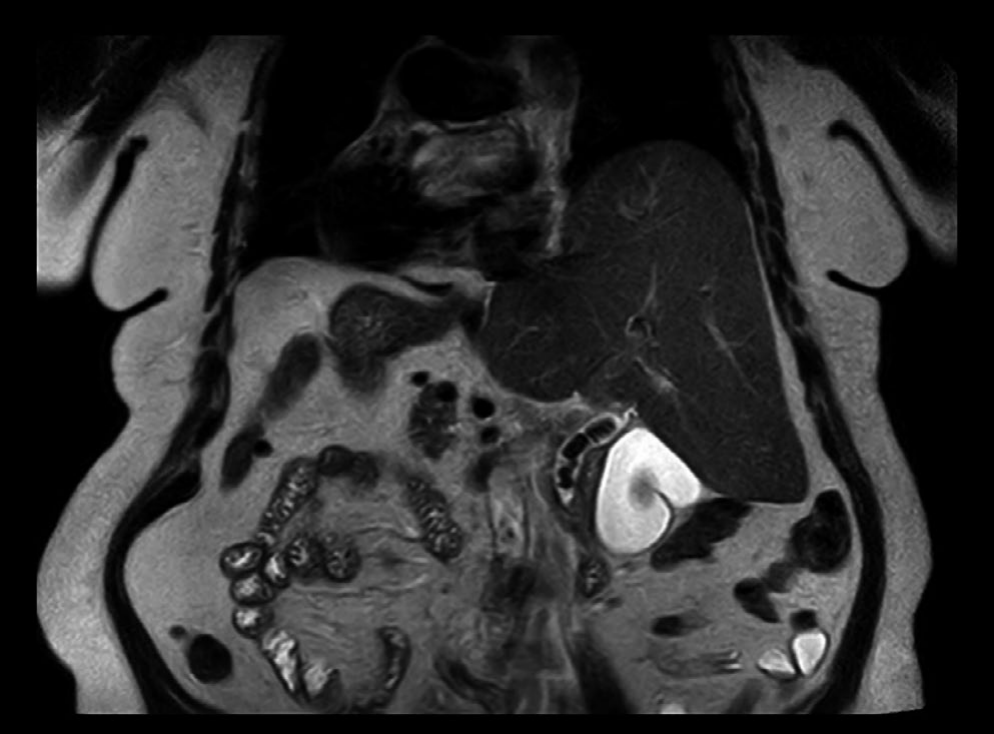
Coronal T2‐weighted MRCP showing multiple common bile duct stones and situs inversus totalis

**Figure 2:**
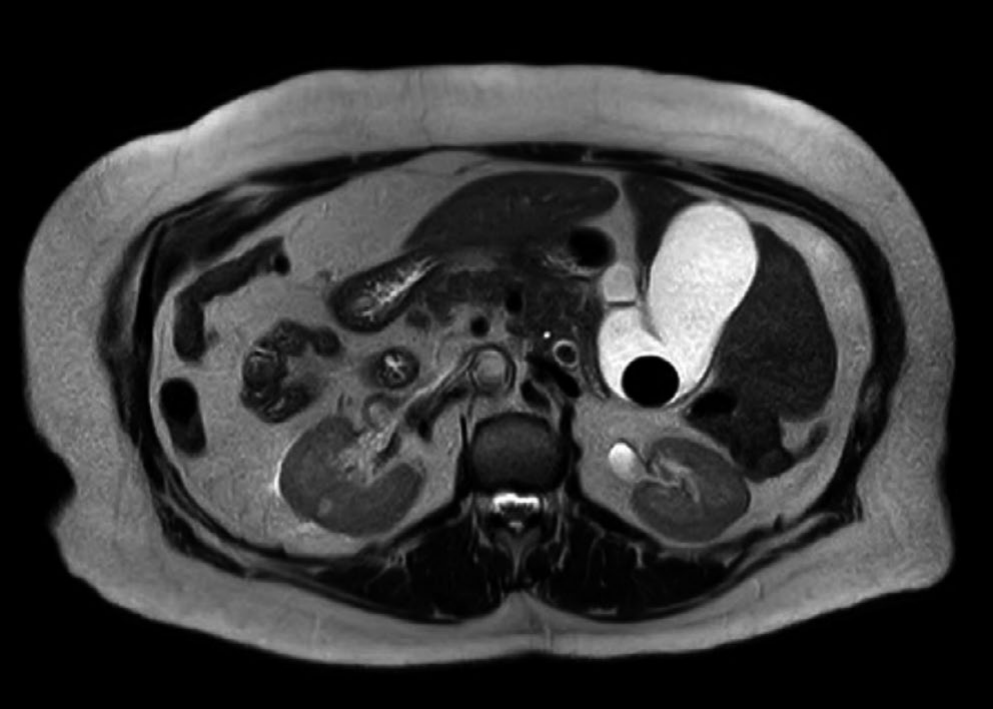
Axial T2‐weighted MRCP showing gallbladder and common bile duct stones with situs inversus

A 79‐year‐old female patient with previous medical history of SIT, hypertension, hypothyroidism, and cholelithiasis presented to the emergency department with a 10‐day history of abdominal pain in the left upper quadrant. In the previous 24 hours, the pain intensified and jaundice and choluria appeared, without fever. At examination, the abdomen was soft, with tenderness in the left upper quadrant. No peritoneal signs were present. Liver function tests showed an elevated total bilirubin (3.09 mg/dL), alkaline phosphatase (437 U/L), and gamma‐glutamyltransferase (1898 U/L). Inflammatory markers were normal. Abdominal ultrasound showed cholelithiasis with a normal diameter common bile duct. Magnetic resonance cholangiopancreatography (MRCP) revealed multiple common bile duct stones (largest stone diameter 15 mm) with dilated common bile duct (14 mm) and SIT findings (Figures 1 and 2).

**Figure 3:**
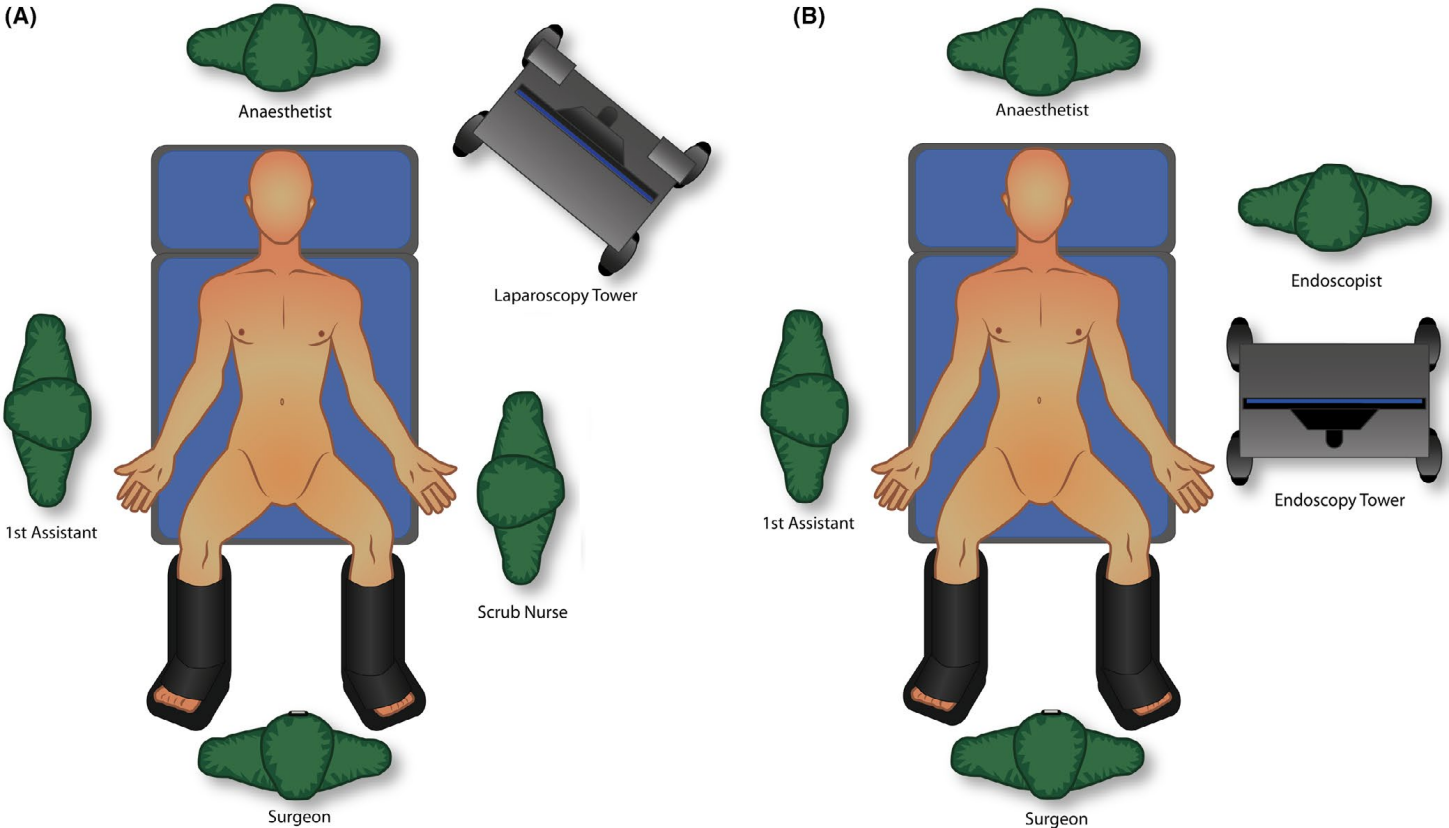
Surgical team position. A, Surgical phase. B, Endoscopic phase

**Figure 4:**
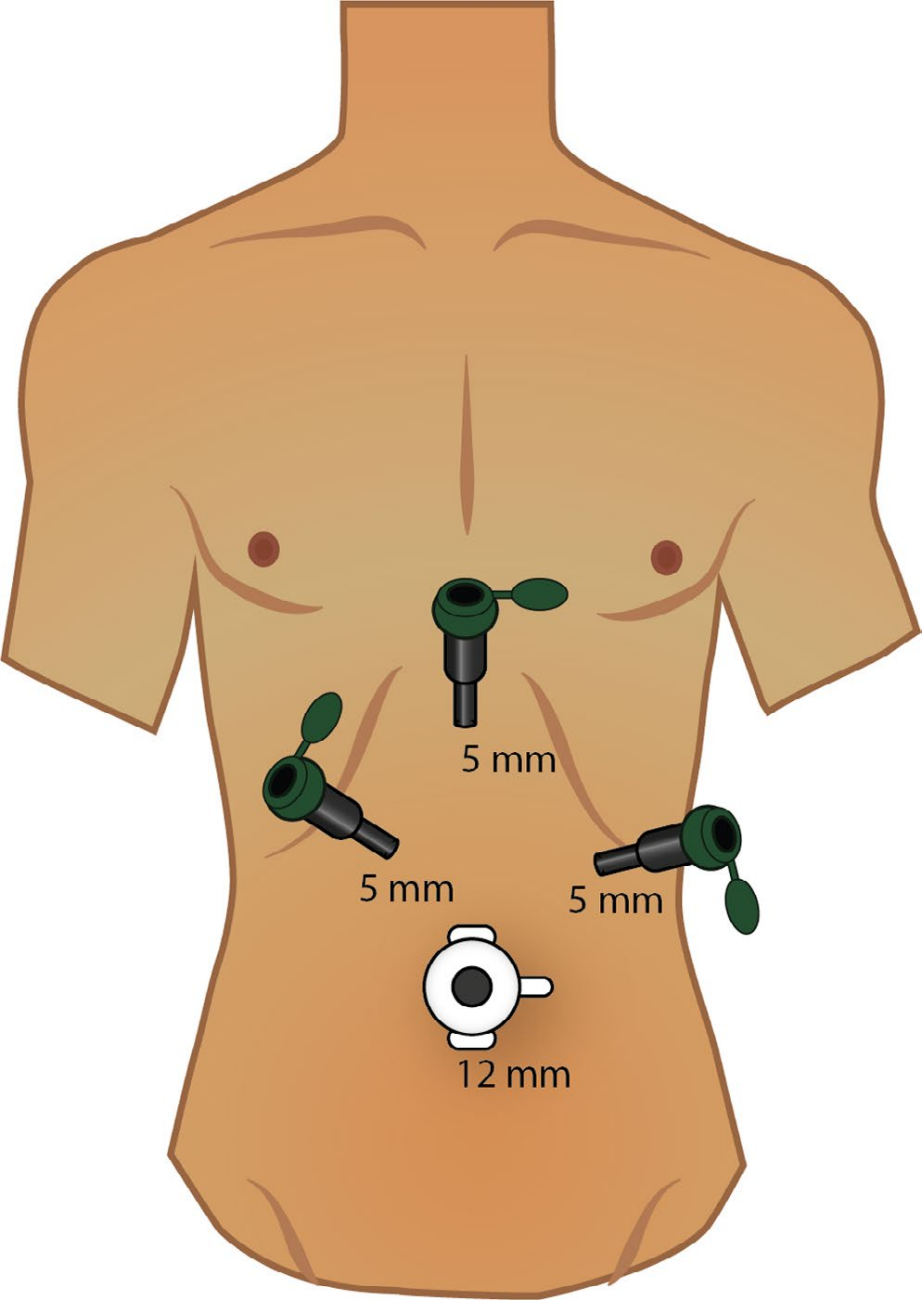
Mirror image French position port placement

**Figure 5:**
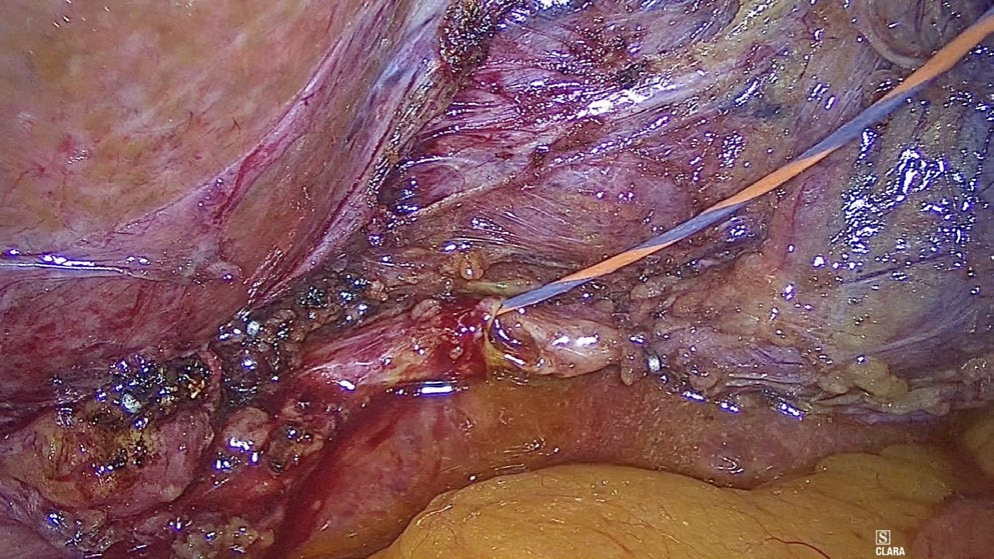
Cystic ductotomy and wire guide inserted through the duct

**Figure 6:**
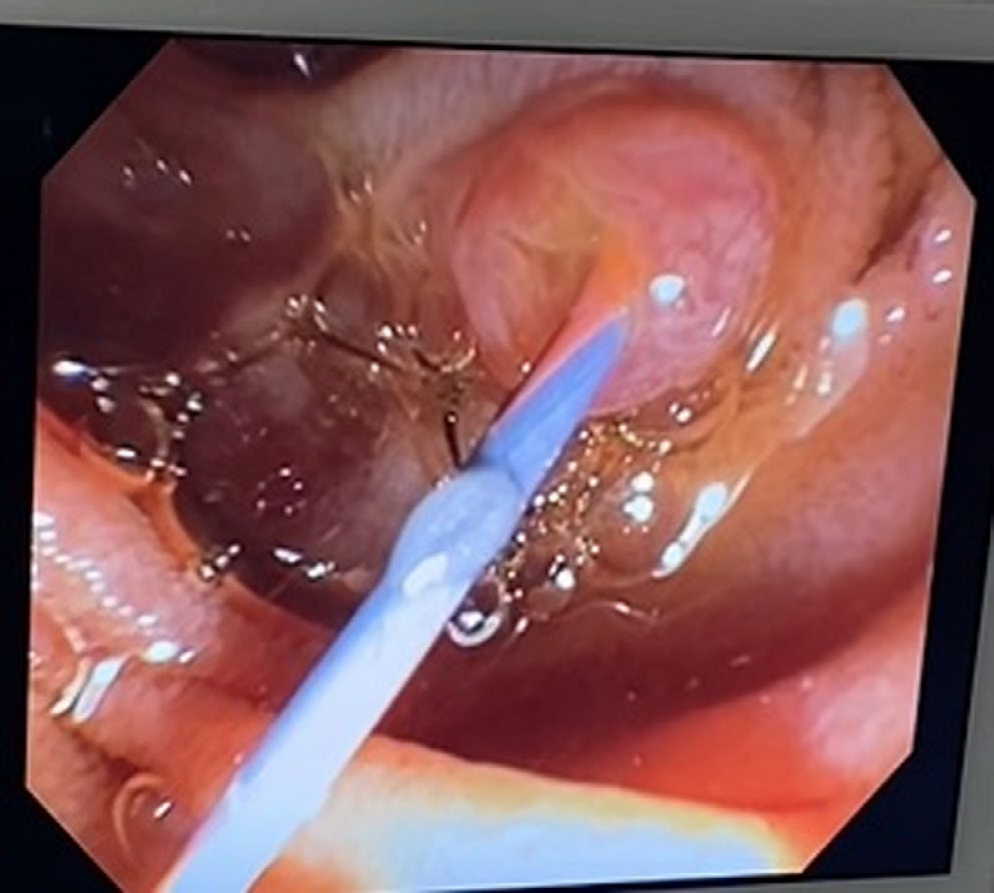
Wire guide is identified through the ampulla by duodenoscopic view

A LER technique was planned to address both pathologies in a one‐stage procedure. The patient was placed in a modified lithotomy position. The surgeon stood between the legs and the first assistant to the right side of the patient. The laparoscopy monitor was placed at the left side of the patient (Figure 3A). One 12‐mm and three 5‐mm trocars were installed in a mirror image French position (Figure 4). Dissection of Calot's triangle was carried out uneventful with the right hand of the surgeon using electrocautery and traction with the left hand in direction to the left side of the patient. A small cystic ductotomy was performed and a cholangiogram was made, confirming multiple common bile duct stones. A wire guide was inserted through the cystic duct (Figure 5), and its position in the duodenum was confirmed by fluoroscopy. The endoscopy tower was positioned at the left side of the patient with the monitor in a cephalad direction and the endoscopist in a caudal direction (Figure 3B). The duodenoscope was advanced through the stomach and duodenum until identification of the wire guide and the ampulla, which was normal (Figure 6). The wire guide was retrieved through the duodenoscope using an extraction basket, and then, a sphincterotome was advanced using the wire. A wide sphincterotomy was made, and extraction of three stones was performed with an extraction balloon. Occlusion cholangiography at the end of the procedure did not show any contrast filling defect suspicious of residual stones. The wire guide was retrieved completely with the duodenoscope. Cholecystectomy continued with double clipping of the cystic duct and cystic artery, and specimen retrieval as usual. The surgical and endoscopic phases were uneventful. The total duration of the complete procedure was 88 minutes.

The patient recovered well after the intervention with normal serum pancreatic lipase and adequate oral intake. Patient was discharged on the first postoperative day. At two‐week follow‐up visit, patient was without complaint.

## 3 DISCUSSION

Situs inversus totalis is a rare condition associated with difficulties in the diagnosis and treatment of several diseases. As the thoracic and abdominal organs are positioned in a mirror image of the normal anatomy, symptoms can be misleading and surgical treatment challenging. LC is the standard of care for the treatment of cholelithiasis, and it has also proven to be a safe method in patients with SIT.^4^ In patients with SIT and cholelithiasis complicated with choledocholithiasis, reported cases have been treated mainly by a two‐stage procedure with LC and preoperative or postoperative ERCP.^5^, ^6^, ^7^, ^8^, ^9^, ^10^ LC + LCBDE as a one‐stage procedure has also been reported.^11^, ^12^, ^13^, ^14^

Even though ERCP has shown to be an adequate treatment with acceptable rates of choledocholithiasis resolution in SIT patients, it is not spared of morbidity and patients may need more than one procedure to achieve a complete common bile duct clearance.^2^ Post‐ERCP pancreatitis (PEP) is the most common complication of this procedure in patients with normal anatomy, with an incidence of 3.5% to 9.7%, and a mortality of 0.1% to 0.7%.^15^ One of the main procedure‐related risk factors for PEP is a difficult bile duct cannulation, with an odds ratio of 1.76‐14.9.^15^ As patients with SIT have a major anatomic variation, it can be inferred that they are at increased risk of PEP as bile duct cannulation may be difficult secondary to the abnormal position. In patients with normal anatomy with cholelithiasis and choledocholithiasis, treatment with one‐stage procedures such as LC + LCBDE and LER has shown to be safe, effective, and associated with lower morbidity, length of hospital stay, and costs compared to two‐stage procedures.^16^, ^17^ LER technique reduces the risk of PEP in a 50%.^18^ Given this evidence, one‐stage procedures may be preferred in patients with SIT and cholelithiasis complicated with choledocholithiasis.

In our case, the modified lithotomy position with mirror image position of the surgical team, trocars, and laparoscopy monitor proved to be comfortable and allowed an uneventful and straightforward procedure with an acceptable total operative time (88 minutes). Although it seems that this procedure is easier if done with the left hand (as a complete mirror image),^4^ we did not encounter major difficulties performing it with the right hand. This depends on the surgeons’ preference. The position of the endoscopy tower at the left side of the patient, with the monitor in a cephalad direction and the endoscopist in a caudal direction, was helpful as the sphincterotomy's sense was the same as in a patient with normal anatomy but in a 1 o'clock direction. This enabled us to perform a wide and safe sphincterotomy, and an uneventful stone extraction. With a one‐day length of postoperative stay, LER proved to be a safe and effective procedure.

Laparoendoscopic rendezvous technique has the complexity of coordinating surgical and endoscopic teams for a one‐stage procedure in the operating room. In our institution, both surgeons and gastroenterologists have ERCP training, so the arrangement of such intervention is easier.

More cases are needed to compare our results. As SIT is a rare condition, prospective trials are unlikely to be done, so we will have to rely on case reports and retrospective studies to draw conclusions.

## 4 CONCLUSION

Laparoendoscopic rendezvous technique may be a feasible procedure in patients with SIT and cholelithiasis complicated with choledocholithiasis. As this condition may confer technical challenges due to the anatomic variations, one‐stage procedures such as LER or LC + LCBDE could be preferred as they may reduce the rate of PEP, overall morbidity and length of hospital stay in the same way as in patients with normal anatomy. In our case, LER achieved resolution of cholelithiasis and choledocholithiasis in a one‐stage procedure with acceptable total operative time, no morbidity, and discharge on the first postoperative day. More cases are needed to compare our results.

## CONFLICT OF INTEREST

None declared.

## AUTHOR CONTRIBUTION

Both authors were involved in substantial contributions to conception and design, data curation and analysis, writing and drafting, and final approval of the version to be published.

## ETHICAL APPROVAL

Published with written consent of the patient.
